# Development of a Risk Assessment Tool to Predict Fall-Related Severe Injuries Occurring in a Hospital

**DOI:** 10.5539/gjhs.v6n5p70

**Published:** 2014-05-13

**Authors:** Shin-ichi Toyabe

**Affiliations:** 1 Crisis Management Office, Niigata University Medical and Dental Hospital,Asahimachi-Dori 1-754, Chuo-Ku, Niigata City, Japan

**Keywords:** falls, risk assessment system, bone fracture, intracranial hemorrhage, adverse events

## Abstract

Inpatient falls are the most common adverse events that occur in a hospital, and about 3 to 10% of falls result in serious injuries such as bone fractures and intracranial haemorrhages. We previously reported that bone fractures and intracranial haemorrhages were two major fall-related injuries and that risk assessment score for osteoporotic bone fracture was significantly associated not only with bone fractures after falls but also with intracranial haemorrhage after falls. Based on the results, we tried to establish a risk assessment tool for predicting fall-related severe injuries in a hospital. Possible risk factors related to fall-related serious injuries were extracted from data on inpatients that were admitted to a tertiary-care university hospital by using multivariate Cox’ s regression analysis and multiple logistic regression analysis. We found that fall risk score and fracture risk score were the two significant factors, and we constructed models to predict fall-related severe injuries incorporating these factors. When the prediction model was applied to another independent dataset, the constructed model could detect patients with fall-related severe injuries efficiently. The new assessment system could identify patients prone to severe injuries after falls in a reproducible fashion.

## 1. Background

Falls are very common adverse events in a hospital and can cause severe injuries. About 3 to 10% of falls in a hospital cause serious injuries such as bone fracture and intracranial hemorrhage (Krauss et al., 2005). These injuries may lead to prolonged length of hospital stay and additional healthcare costs. A previous study showed that the operational cost for fallers with serious injuries was $13 316 more than that for controls and that fallers stayed 6.3 days longer than non-fallers (Wong et al., 2011). This situation results in psychological distress for and complaints from the patients with possible litigation from the patients and their families.

A strategy to prevent inpatient falls is a target prevention strategy by selecting patients at high risk for falls (Gates, Fisher, Cooke, Carter, & Lamb, 2008). Several clinical characteristics have been shown to be associated with increased incidence of falls in a hospital, and various risk assessment tools for inpatient falls have been developed through integration of these risk factors. However, these tools were developed to find patients at high risk for falls and were not designed to predict patients who would suffer physical injuries after falls (Oliver, Daly, Martin, & McMurdo, 2004; Papaioannnou et al., 2004; Oliver et al., 2008; Kim, Mordiffi, Bee, Devi, & Evans, 2007; Heinze, Dassen, Halfens, & Lohrmann, 2009). In reality, the STRATIFY (St. Thomas Risk Assessment Tool in Falling elderly inpatients) tool (Oliver, Britton, Seed, Martin, & Hopper, 1997) rated half of the patients who fractured as low risk of falling in Japanese acute care hospital setting (Tayabe, 2010). The STRATIFY (St. Thomas Risk Assessment Tool in Falling elderly inpatients) tool is commonly used as a fall risk assessment tool in clinical practice. One of the most important reasons for preventing falls is to prevent serious injuries in patients at high risk for injuries after falls (Gates, 2008). Risk assessments tools are needed to predict falls that are likely to be complicated with serious injuries.

We previously reported that the most frequent serious injury after falls was bone fracture and the second most frequent serious injury was intracranial hemorrhage and that these two kinds of injuries accounted for almost all severe injuries after falls (Toyabe, 2010, 2012). We further found that not only bone fractures after falls but also intracranial hemorrhage after falls were significantly associated with the FRAX™ risk assessment score for osteoporotic bone fracture. Occurrence of intracranial hemorrhage was significantly more associated with the FRAX™ score than co-existing hemorrhagic disorders and administration of anticoagulants or anti-platelets. The fact that both of the two major fall-related injuries were associated with FRAX™ score suggest the possibility to construct a risk assessment model to predict severe injuries after falls by incorporating the FRAX™ score. On the basis of these findings, we tried to construct risk assessment models to predict serious injuries after falls by integrating various identified risk factors including risk assessment scores for falls and bone fractures. We then selected the most appropriate model by comparing the performances of the constructed prediction models and validated the performance of the model by using another independent dataset.

## 2. Methods

### 2.1 Settings

This study was conducted at Niigata University Hospital, an 825-bed academic hospital in the city of Niigata. There are 23 clinical departments and the service area of the hospital as a tertiary care hospital covers all districts in Niigata Prefecture, which has a population of 2 400 000.

### 2.2 Study Subjects

The development dataset that was obtained from all patients who had been admitted to the hospital during the period from April 2006 to March 2010 and who were aged from 40 to 90 years at admission was used to construct models to predict fall-related severe injuries. During that period, 29 770 patients were admitted to the hospital, but 660 patients were excluded from the study because of missing data. Finally, data were obtained from 29 110 patients (696 365 patient-days) including 13 945 females and 15 165 males.

### 2.3 Data Collection

Information on the patients’ background was obtained from a data warehouse of the hospital information system. The data warehouse includes data for gender, age, body weight, height, history of bone fractures, smoking habit, alcoholic consumption, prescriptions of various drugs, coexisting illness, admission day, discharge day, background disease based on ICD-10 codings, admission ward, and diagnosis and treatment department. Among the drugs prescribed for the patients, we focused on ‘culprit’ drugs for falls and drugs that may cause hemorrhagic tendency. The ‘culprit’ drugs for falls include centrally sedating agents (sedatives, hypnotics, opiates and anticonvulsants) and drugs that can precipitate postural hypotension, arrhythmia or syncope (antihypertensives, diuretics and antiarrhythmics). The drugs that may cause hemorrhagic tendency include anticoagulants and antiplatelets. Information on risk factors for falls was obtained from medical charts of the patients and fall assessment records completed by attending nurses at admission. The medical charts and assessment records included information on history of falls, gait instability, agitated confusion, urinary incontinence or frequency, visual impairment, lower limb weakness and prescription of ‘culprit’ drugs for falls.

### 2.4 Risk Assessment Tools for Falls and Fractures

We calculated the STRATIFY score and the FRAX™ score for each admission of each patient by using data that was obtained by above-mentioned method. The STRATIFY score was used to assess patients’ risk for falls in our study. The STRATIFY score is based on five factors: history of falls, agitated confusion, visual impairment, urinary frequency and high transfer/mobility score (Oliver, 1997). A score of more than two was considered high risk for falls in a Japanese setting, when the score was calculated on the basis of the original method (Toyabe, 2010). The FRAX™ score was proposed by the World Health Organization to compute ten-year probability of osteoporotic fracture by integrating various risk factors for osteoporosis (Fujiwara et al., 2008; Kanis et al., 2005; WHO, 2008). These risk factors include age, prior fragility fracture, parental history of hip fracture, smoking, use of systemic corticosteroids, excess alcohol intake and rheumatoid arthritis. The FRAX™ score was calculated according to body mass index on the basis of the ten-year probability of major osteoporotic fracture in the Japanese population.

### 2.5 Falls and Severe Injuries after Falls

Data on fall events were obtained from online incident reports and from text data of image order entries (Toyabe, 2012). Medical staff who find inpatients who have fallen are encouraged to report the events by using an online intra-institutional incident reporting system. The incident reports contain information on degree of injury, potential causative factors of the incident, type of events and essential information on the event such as the name of the patient involved in the event, the name of the medical staff involved, the exact time and place, detailed description of the course of the event, action against the event taken by medical staff and outcome of the event. It is easy to identify fall-related reports among all reports according to information on the category of reports. When the physician who is responsible for the fallen patient finds signs or symptoms that suggest severe injuries such as bone fracture or intracranial hemorrhage, the physician orders an x-ray examination of the affected area or computed tomography scan of the head through image order entries. Therefore, text data of image order entries are expected to contain information on fall-related severe injuries in a more concentrated manner compared with incident reports and to contain information on fall events that are not reported in incident reports. The text data of image order entries contain information on possible diagnosis, short clinical course and purpose of the order. Severe injuries after falls correspond to cases in which the degree of harm is moderate, serious or fatal in terms of the framework of the international classification of patient safety, and they include bone fractures and intracranial hemorrhage (WHO, 2009). Pain, bruises, isolated hematomas and superficial wounds were excluded from severe injuries. Peripheral bone fractures were included only when they were verified by radiographic examination. Vertebral compression fractures were included only when they were not detected by radiographic examination before the falls but were first detected by radiographic examination after falls. Diagnosis of intracranial hemorrhage was made by a computed tomography scan or magnetic resonance imaging.

### 2.6 Detection of Risk Factors for Severe Injuries after Falls

We tried to detect significant risk factors for severe injuries after falls from analysis of the development dataset by using two different methods. The first method was the difference in proportions test and multiple logistic analyses in which time between admission and falls was not considered in the analysis. In multiple logistic analyses, significant risk factors were detected by using the stepwise selection method. The second method was survival analyses in which time between admission and falls was considered as survival time. The reason why we used survival analysis is that length of stay in acute care hospitals in Japan is very long compared with that in other countries, and the length of hospital stay should affect frequency of inpatient falls (OECD, 2012). Discharge from the hospital without falls was considered as censoring. The Kaplan-Meier method was used for the analyses, and the logrank test was used to examine whether each risk factor was significantly associated with falls. The multivariate Cox’ s proportional hazards model was used to examine risk factors that were most significantly associated with falls among the various risk factors. The stepwise selection method was used to select significant risk factors in the multivariate analyses. Spearman’ s rank coefficient was used to determine if there were associations between the selected risk factors.

### 2.7 Construction of Risk Assessment System for Severe Injuries after Falls

The models for risk assessment of severe injuries after falls were constructed by incorporating the significant risk factors using three different methods. In the first method, each risk factor was scored on the basis of the regression coefficient that was obtained by multivariate Cox’ s proportional regression analysis. The regression coefficients were divided by the smallest coefficient and then rounded to the nearest integer. Each individual risk score was added to form a total risk score for severe injuries after falls. The cut-off value to differentiate high and low risks for severe injuries after falls was determined on the basis of the Youden index from the receiver operating characteristics (ROC) curve. In the second method, each risk factor was scored on the basis of the regression coefficient of multivariate logistic regression analyses and the subsequent procedure was the same as that in the first method. In the third method, cut-off values were set for all significant factors based on the Youden index from the ROC curve. When all significant factors exceed their cut-off values, the patient was considered as being at high risk for severe injuries after falls. The three models were applied to the development dataset, and the most appropriate model was selected in terms of sensitivity, specificity, positive predictive value (PPV), negative predictive value (NPV) and F-measure. F-measure is a harmonic mean of sensitivity and PPV.

### 2.8 Validation of the Risk Assessment Tool by the Test Dataset

Post-hoc power analysis was performed to estimate sample power of the analysis of the development. Based on the results, we estimated necessary sample size for test dataset that was used to validate the selected model for the risk assessment tool. The selected model was then applied to the test dataset to ascertain whether the results obtained from analyses of the development dataset were reproducible. Sensitivity, specificity, PPV, NPV and F-measure were calculated. The incidences of severe injuries after falls were compared in high-risk and low-risk patients using the differences in proportions test and logrank test.

### 2.9 Statistical Analysis and Ethical Consideration

All data were analyzed anonymously. Statistical analyses were performed using IBM SPSS statistics (SPSS Japan Inc., Tokyo, Japan) and Stata/SE 11.2 (StataCorp LP, TX, USA). A p-value less than 0.05 was considered statistically significant. The Ethics Committee of Niigata University School of Medicine gave ethical approval of the study (No. 1666).

## 3. Results

### 3.1 Severe Injuries after Falls

Among the 29 110 patients (696 365 patient-days) for whom data in the development dataset were used for analyses, 47 patients experienced severe injuries after falls. Rate of occurrence of severe injuries after falls was calculated as 0.067/1 000 patient-days. The injuries included bone fractures (33 cases, 70.2%) and intracranial hemorrhage (11 cases, 23.4%). The other three cases were disruption of surgical wounds, rupture of a liver tumor and facial laceration with fracture of the teeth. These three cases were excluded from subsequent analyses because number of patients who belonged to these categories of injury was small. Therefore, the 44 cases were considered as cases of fall-related severe injuries in the development dataset. Bone fractures included 28 cases of peripheral bone fracture (59.6%) and 5 cases of vertebral compression fracture (10.6%).

### 3.2 Factors Associated with Fall-Related Severe Injuries

We tried to find factors that are closely associated with severe injuries after falls by using the development dataset. Univariate analysis revealed that a past history of falls, age, gender, agitated confusion, frequent urination, lower limb weakness, LOS, administration of any anticoagulants and/or antiplatelets, warfarin administration, STRATIFY score and FRAX™ score were significantly associated with fall-related severe injuries both by the logrank test and differences in proportions test (Table 1). Multivariate Cox’s regression analysis with the stepwise selection method revealed that STRATIFY score and FRAX™ score were significantly associated with fall-related severe injuries among the various possible risk factors (Table 2A). Similarly, multiple logistic regression analysis revealed that STRATIFY score and FRAX™ score were significantly associated with severe injuries after falls (Table 2B). Therefore, we constructed models to predict severe injuries after falls by incorporating the STRATIFY score and the FRAX™ score into the models. The cut-off value of the STRATIFY score to predict falls was determined to be a value of 2 based on the results of ROC analysis of data in the development dataset. The cut-off value of FRAX™ score to predict bone fractures after falls was determined to be a value of 10 based on the results of ROC analysis of data in the development dataset. There was no significant association between the STRATIFY score and the FRAX™ score (Spearman’ s rank coefficient r=0.207).

**Table 1:** Results of univariate analysis of risk factors for severe injuries after falls

Factors	Fallers with severe injuries	Non-fallers and fallers without severe injuries	Logrank test	Chi-square test
All patients	43	29 067		
History of falls	27	12 176	0.003	0.008
Age >=65	34	15 283	<0.001	<0.001
Male gender	13	15 305	0.002	0.005
BMI >=30	6	6 777	0.465	1.000
Gait instability	8	2 223	0.121	0.016
Agitated confusion	11	1 823	0.003	<0.001
Frequent urination	10	2 352	0.010	<0.001
Visual impairment	11	5 093	0.097	0.235
Lower limb weakness	19	4 814	0.005	<0.001
Prescription of ‘culprit’ drugs	9	3 338	0.656	0.089
STRATIFY >=2	23	6 372	<0.001	<0.001
FRAX™ >=10	32	10 438	<0.001	<0.001
LOS >=14	31	14 115	-	0.003
Ward	-	-	0.636	<0.001
Clinical department	-	-	0.815	<0.001
Background disease (ICD10)	-	-	0.086	<0.001
Any anticoagulants or antiplatelets	19	4 846	0.008	<0.001
Warfarin	13	2 606	0.010	<0.001

Factors that may associated with falls and severe injuries after falls were evaluated to determine whether they are associated with severe injuries after falls by using the differences in proportions test and logrank test in the development dataset. LOS, length of hospital stay.

**Table 2:** Results of multivariate analysis of risk factors for severe injuries after falls

A.						
Items	Estimated coefficient (β)	Standard error for β	Sig.	Hazard ratio	95% C.I. for β	
STRATIFY	0.431	0.121	<0.001	1.539	1.214	1.952
FRAX™	0.048	0.012	<0.001	1.049	1.024	1.074

B.						
Items	Estimated coefficient (β)	Standard error for β	Sig.	Odds ratio	95% C.I. for β	
STRATIFY	0.539	0.118	<0.001	1.714	1.359	2.161
FRAX™	0.052	0.012	<0.001	1.054	1.029	1.079
Constant	-7.780	0.284	<0.001			

Risk factors that were significantly associated with severe injures after falls in Table 1 were analyzed by multivariate Cox’ s regression analysis (A) and multiple logistic regression analysis (B). Significant factors were selected by using the stepwise selection method. CI, confidence interval.

Factors that may associated with falls and severe injuries after falls were evaluated to determine whether they are associated with severe injuries after falls by using the differences in proportions test and logrank test in the development dataset. LOS, length of hospital stay.

Risk factors that were significantly associated with severe injures after falls in Table 1 were analyzed by multivariate Cox’ s regression analysis (A) and multiple logistic regression analysis (B). Significant factors were selected by using the stepwise selection method. CI, confidence interval.

After three models had been constructed to predict severe injuries after falls, we compared the performances of the models by applying the models to the development dataset (A) and the test dataset (B). Sensitivity, specificity, positive predictive value (PPV), negative predictive value (NPV) and F-value were calculated. Event +ve, event positive; event -ve, event negative.

### 3.3 Construction of Models to Predict Fall-Related Severe Injuries

Three models to predict severe injuries after falls were constructed. The first model was constructed on the basis of the regression coefficients of the two risk factors obtained by multivariate Cox’s regression analysis. To determine the weight for each risk factor, the regression coefficients were divided by the smaller coefficient and rounded to the nearest integer (Table 2A). After adding each risk score to form total risk score, the cut-off value to differentiate high risk and low risk for severe injuries after falls was determined on the basis of the Youden index from the ROC curve. As a result, total risk score exceeding a value of 55 was considered as being high risk for severe injuries after falls. In the second model, the weight for each risk factor was similarly obtained from the results of multivariate logistic regression analysis (Table 2B). The cut-off value in the second model was determined to be a value of 33. In the third model, patients with a FRAX™ score of more than the cut-off value of 10 and with a STRATIFY score of more than the cut-off value of 2 were considered to be at high risk for severe injuries after falls.

### 3.4 Performance of the Predictive Models

The three models were applied to the development dataset to select the most appropriate model to predict fall-related severe injuries (Table 3A). Since the frequency of falls with severe injuries in the hospital was low, the value of PPV and the F-value were very small for all of the models or risk assessment tools used. Among the three models, model 2 and model 3 were the best in terms of F-value. On the other hand, specificity was excellent in model 3 followed by model 2. Therefore, we considered model 3 to be the most appropriate predictive model for severe injuries after falls.

**Table 3:** Comparison of performances of the constructed models to predict severe injuries after falls A.Development dataset

Model	Risk Criteria	Event +ve	Event -ve		Total		Sensitivity (%)	Specificity (%)	PPV (%)	NPV (%)	F-measure
1	High	26	5 205		5 231						
	Low	17	23 862		23 879		60.47	82.09	0.50	99.93	0.0099
	Total	43	29 067		29 110						

2	High	25	4 282		4 307						
	Low	18	24 785		24 803		58.14	85.27	0.58	99.93	0.0115
	Total	43	29 067		29 110						

3	High	18	3 118		3 136						
	Low	25	25 949		25 974		41.86	89.27	0.57	99.90	0.0113
	Total	43	29 067		29 110						

STRATIFY	High	23	6 372		6 395						
	Low	20	22 695		22 715		53.49	78.08	0.36	99.91	0.0071
	Total	43	29 067		29 110						

FRAX™	High	32	10 438		10 470						
	Low	11	18 629		18 640		74.42	64.09	0.31	99.94	0.0061
	Total	43	29 067		29 110						

No screening		43	29 067		29 110				0.15		

B. Test dataset											

Model	Risk Criteria	Event +ve	Event -ve		Total		Sensitivity (%)	Specificity (%)	PPV (%)	NPV (%)	F-measure
1	High	14	4 685		4 699						
	Low	4	14 902		14 906		77.78	76.08	0.30	99.97	0.0059
	Total	18	19 587		19 605						

2	High	14	3 879		3 893						
	Low	4	15 708		15 712		77.78	80.20	0.36	99.97	0.0072
	Total	18	19 587		19 605						

3	High	9	2 337		2 346						
	Low	9	17 250		17 259		50.00	88.07	0.38	99.95	0.0076
	Total	18	19 587		19 605						

STRATIFY	High	10	3 327		3 337						
	Low	8	16 260		16 268		55.56	83.01	0.30	99.95	0.0060
	Total	18	19 587		19 605						

FRAX™	High	17	10 522		10 539						
	Low	1	9 065		9 066		94.44	46.28	0.16	99.99	0.0032
		Total	18	19 587		19 605						

No screening		18	19 587		19 605				0.09		

After three models had been constructed to predict severe injuries after falls, we compared the performances of the models by applying the models to the development dataset (A) and the test dataset (B). Sensitivity, specificity, positive predictive value (PPV), negative predictive value (NPV) and F-value were calculated. Event +ve, event positive; event -ve, event negative.

### 3.5 Validation of the Constructed Model

**Figure 1:**
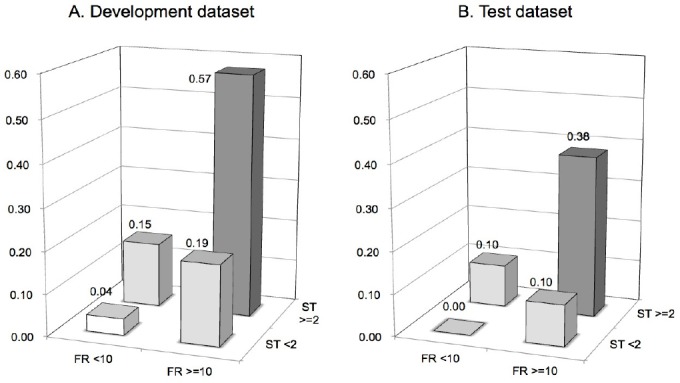
Two-dimensional risk assessment matrix composed of FRAX™ score and STRATIFY score

**Figure 2:**
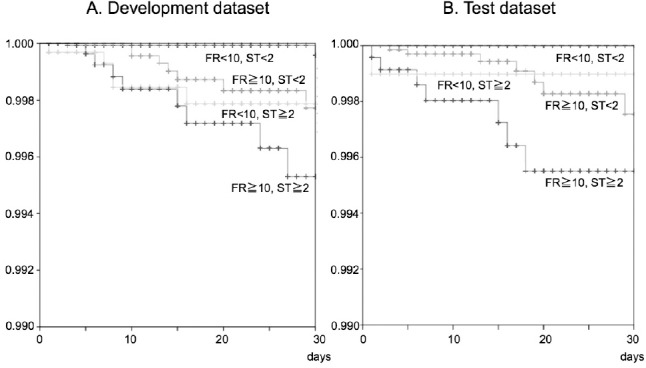
Survival plots for severe injuries after falls

Post-hoc power analysis revealed that statistical power of the analysis of the development dataset was estimated as 0.997 in the difference in proportions test and as 0.998 in the logrank test, respectively. When we tried to detect the significant results under the same sample power and significance level of 0.001, necessary sample size for test dataset was calculated as at least 14 000 in the difference in proportions test and 18 000 in logrank test. To get enough sample size, we used data obtained from all patients who had been admitted to the hospital during the period from April 2010 to March 2012 and who were aged from 40 to 90 years at admission. During that period, 19 931 patients were admitted to the hospital, but 326 patients were excluded from the study because of missing data. Finally, data were obtained from 19 605 patients (390 370 patient-days) including 10 626 females and 8 979 males, which yield necessary number of samples with respect to both the difference in proportions test and logrank test. Eighteen patients experienced severe injuries after falls among these patients, and rate of occurrence of severe injuries after falls was calculated as 0.046/1 000 patient-days. All of the severe injuries were bone fractures, and they included 15 cases of peripheral bone fracture (83.3%) and 3 cases of vertebral compression fracture (16.7%). When model 3 was applied to the test dataset, this prediction model again showed good results. As shown in Table 3B, performance of model 3 was superior to that of other models in terms of F-measure and specificity. Model 3 was a two-dimensional risk assessment tool that consisted of the FRAX™ score and the STRATIFY score. Figure 1 shows two-dimensional risk assessment matrices, and the frequency of fall-related severe injuries in each matrix is shown in a bar graph. The frequency of fall-related severe injuries was highest in patients with both FRAX™ and STRATIFY scores that exceeded their cut-off values (double positive group). The frequency of fall-related severe injuries in this matrix was significantly higher than that in the other matrices both by the differences in proportions test (p<0.001) and log-rank test (p<0.001). Patients for whom one of the two assessment scores exceeded the cut-off value showed the next highest frequency of injurious falls (single positive group), and patients with two scores that were less than their cut-off values showed the lowest frequency of fall-related severe injuries (double negative group). There were significant differences in frequencies of serious injuries among the double positive group, single positive group and double negative group. Kaplan-Meier analysis and the logrank test revealed that there were significant differences in occurrence of fall-related severe injuries among the double negative group, the double positive group and the patient group in which only the FRAX™ score exceeded the cut-off value (Figure 2). There was no significant difference between the double negative group and the patient group in which only the STRATIFY score exceeded the cut-off value.

Patients belonging to development dataset (A) and test dataset (B) were divided into four groups by determined cut-off values of FRAX™ score and STRATIFY score. The bar graph shows that the percentage of patients with fall-related severe injuries in each group.

All patients were plotted on the Kaplan-Meier survival curve as a function of hospital length of stay. Cumulative rates of fall-related severe injuries were compared between patients belonging to the four groups as shown in Figure 1.

## 4. Discussion

In the present study, we examined a new assessment system to evaluate the risk of severe injuries after falls. This risk assessment system uses the combination of a risk assessment score for inpatient falls and a risk assessment score for osteoporotic bone fracture. We found in our previous study that the FRAX™ risk assessment score for osteoporotic bone fracture was significantly associated not only with bone fractures after falls (Toyabe, 2010) but also with intracranial hemorrhage after falls (Toyabe, 2012). Since almost all of the fall-related severe injuries were bone fractures and intracranial hemorrhage, it is reasonable for the FRAX™ score to be significantly associated with fall-related severe injuries as a whole. We found that the combined risk assessment system could identify patients prone to severe injuries after falls in a reproducible manner.

A strategy to reduce inpatient falls as well as physical injuries after falls is a target prevention strategy by selecting patients at high risk for falls using various risk assessment systems for falls (Gates, 2008). However, these systems were originally developed to find patients at high risk for falls and were not designed to find patients prone to severe injuries after falls (Oliver, 2004, 2008; Papaioannou, 2004; Kim, 2007; Heinze, 2009). The risk assessment tools for falls do not have sufficient performance to predict physical injuries after falls. In fact, only 53.4% of the patients with serious fall injuries in the development dataset and only 55.6% of the patients with serious fall injuries in the test dataset were judged as being at high risk for falls before they had been affected by fall injuries. Severe injuries after falls are the most important issue in inpatient falls because these injuries may lead to prolonged length of stay and psychological distress for the patients and additional healthcare costs. Cost attributable falls are highly skewed to those that result in physical injuries (Wu, Keeler, Rubenstein, Maglione, & Shekelle, 2010). Some authors expressed an opinion that prevention strategies should focus on fall injuries rather than falls per se (Quigley et al., 2009). Risk assessment tools are needed to detect fallers that are likely to be complicated with severe injuries.

The risk assessment system for fall-related severe injuries that we used in this study incorporates two risk assessment scores for bone fractures and for inpatient falls. We previously reported that the STRATIFY score was useful for prediction of inpatient falls in a Japanese acute care hospital setting in spite of its performance not being optimal (Toyabe, 2010). A systematic review showed that the diagnostic accuracy of the STRATIFY rule is limited and should not be used in isolation for identifying individuals at high risk for falls. According to this opinion, it might be reasonable to use multiple risk assessment systems to evaluate the risk for falls with severe injuries. In another risk assessment system, FRAX™, fall-risk related factors are not incorporated into the FRAX™ model. However, we previously reported that a high FRAX™ score was significantly associated with inpatient falls (Toyabe, 2010). Considering that there was no significant association between the STRATIFY score and FRAX™ score, the FRAX™ score might detect a group of fall-prone patients who have different characteristics from those who showed high STRATIFY scores.

The results of this study showed that patients with FRAX™ and STRATIFY scores exceeding their cut-off value (double positive group) suffer serious injuries more frequently than do other patient groups. Patients in whom one of the two assessment scores exceeded the cut-off value showed the next highest frequencies of injurious falls, and there were significant differences in the frequency of injurious falls among the double positive group, single positive group and double negative group. If we consider only the double positive group as a high-risk group for fall injuries, we might overlook the risk of fall-related severe injuries that occur in the single positive group. The single positive group should be considered as the next high-risk group for injurious falls. Further investigation is needed to determine how to differentiate interventions to prevent injurious falls for patients belonging to the double positive group and for patients belonging to the single positive group.

There have been many studies on risk factors for fall-related injuries, but no consensus regarding the risk factors for injuries after falls has been reached. Furthermore, there have been no studies on a risk assessment system of injurious falls. Fisher et al. reported that patients 75 years or older and patients on the geriatric psychiatry floor were more likely to sustain serious fall-related injuries (Fischer et al., 2005). Krauss et al. reported that advanced age, falls in the bathroom and unassisted falls were associated with injury (Krauss et al., 2007). Bradley et al. reported that trauma after falls and ambulatory status were predictors of injury (Bradley, Karani, McGinn, & Wisnivesky, 2010). Milon et al. reported that patients who were administered some kinds of drugs were more likely to sustain an injuries (Milon et al., 2012). Some of these possible risk factors such as advanced age were in accord with the results of our study, but we could not find any significant association between injurious falls and most of these possible risk factors.

There are several limitations of this study. First, we limited the study subjects to patients aged from 40 to 90 years. This was because the FRAX™ system targets that age group. Second, this was a single institute study. Since some fall characteristics differed by hospital size and type, a multicenter study is necessary to validate whether the assessment tool that we developed is useful for other acute care hospitals. Third, a considerable number of fall events might be missed by the incident reporting system. Underreporting or non-reporting is an inevitable issue of this method because the method relies on voluntary willingness of individuals. However, the number of missed cases was thought to be small because we focused on severe injuries after falls rather than fall events themselves. It is difficult to miss any patients with severe injuries after falls. In addition, we collected data on severe injuries after falls not only from incident reports but also from information on image order entries. Since diagnostic imaging is necessary to assess injuries after falls, it is unlikely we missed the cases with severe injuries after falls.

## 5. Conclusions

By integrating a risk assessment score for inpatient falls and the risk assessment score for osteoporotic bone fracture, we developed a new assessment system to evaluate the risk of severe injuries after falls. The combined risk assessment system could identify patients prone to fall-related severe injuries, and risk assessment by this system was valid in a reproducible fashion.
